# The Risk of Substance Use Among Adolescents and Adults With Eating Disorders

**DOI:** 10.7759/cureus.10309

**Published:** 2020-09-08

**Authors:** Noha Eskander, Sumita Chakrapani, Mohammad R Ghani

**Affiliations:** 1 Psychiatry, California Institute of Behavioral Neurosciences & Psychology, Fairfield, USA; 2 Neurology, California Institute of Behavioral Neuroscience & Psychology, California, USA

**Keywords:** child and adolescent psychiatry, anorexia nervosa, eating behaviors, psychology, substance induced disorders, bulimia nervosa, alcohol misuse, addiction psychiatry, public psychiatry, psychiatry & mental health

## Abstract

Eating disorders (EDs) are negative eating habits that have harmful mental and physical effects. EDs primarily affect young women. Most cases are diagnosed in adolescence. The most common EDs are bulimia nervosa (BN), anorexia nervosa (AN), and binge eating disorder (BED). There is a strong association between EDs and substance use disorder (SUD) in adolescence. Bulimia nervosa and alcohol use disorder (AUD) are the most common co-occurrence. There is a high behavioral association between EDs and AUD. Alcohol consumption could be a primary trigger for binge eating in women with BN. Alcohol can be used as an appetite suppressor and as a compensatory behavior to avoid food.

The objective of this literature review was to explore the relation between EDs and SUD. The results of the study showed SUD is common with EDs. There are many reasons for this association such as shared neurobiological mechanisms, personality features, environmental and genetic factors.

## Introduction and background

Eating disorders (EDs) are negative eating habits that have adverse mental and physical effects. EDs primarily affect young women who are mostly diagnosed in adolescence and early adulthood. Only 10% of all cases of EDs are among men [1]. The most common EDs are anorexia nervosa (AN), bulimia nervosa (BN), and binge eating disorder (BED). The lifetime prevalence rate of EDs is low with 0.1% to 1% in AN and 1% to 3% in BN [1]. Women with AN have a strong desire to be thin so they restrict food and fear gaining weight. They often perceive themselves as overweight when in fact they are underweight. They try to achieve their weight loss through over-exercising, using laxatives, restriction of caloric intake, diuretics, and purging [2].

Bulimia nervosa is characterized by multiple, recurrent episodes of eating a large amount of food (binging) associated with loss of control followed by inappropriate compensatory behavior such as excessive exercise, fasting, self-induced vomiting, and misuse of laxatives or diuretics [3]. According to the *Diagnostic and Statistical Manual of Psychiatry - Fifth Ddition* (DSM-5), these episodes must happen at least once per week for a three month period [3].

Binge eating disorder is the most common eating disorder. It affects 3% of the general population in the United States [4]. It is characterized by binge eating episodes once or more per week for three months with the episodes lasting two or more hours. These episodes are psychologically distressful during which people with BED consume a large amount of food. These episodes are also associated with a sense of loss of control [4].

Substance use disorder (SUD) typically starts in adolescence and early adulthood. The prevalence of SUD is 30% among adults in the United States [5]. Approximately 9% of adolescents have a drug-use disorder and 6% have an alcohol use disorder (AUD) [5].

There is a strong association between EDs and SUD in adolescence. The most common co-occurrence is BN and AUD. The National Center on Addiction and Substance Abuse mentioned that approximately 50% of women with EDs have SUD [6]. Similarly among those with SUD, the rates of EDs are high. There are increased rates of AUD among those with SUD. It was reported that there is a direct association between the number of drinks on one occasion and the risk of having or developing EDs [7]. Adolescents who struggle with binge eating are also more likely to report drinking more at each occasion and getting drunk than those who do not binge eat [7].

In this literature review, we aimed to investigate the link between EDs and SUD. In this study we will investigate the reasons and the risk factors behind this comorbidity. This study also aims to understand the socio-demographic, behavioral associations, psychopathology, and the psychosocial outcome in adolescents and adults with these two disorders.

## Review

Methods and results

Data was searched on Pub Med using regular keywords “Eating Disorders”, “Substance Use Disorder”, “Anorexia Nervosa”, "Bulimia Nervosa", “Binge Eating Disorder”, and “Alcohol Use Disorder”. Table 1 shows the results of the search.

**Table 1:** PubMed keywords search results

Keywords	Database	Date	Number of results
Eating Disorders	Pub Med	8/14/2020	43,611
Substance Use Disorder	Pub Med	8/14/2020	290,129
Anorexia Nervosa	Pub Med	8/14/2020	16,720
Bulimia Nervosa	Pub Med	8/14/2020	7,716
Binge Eating Disorder	Pub Med	8/14/2020	4,463
Alcohol Use Disorder	Pub Med	8/14/2020	106,541

Results

The total number of keywords search results was 469,180. The following is a breakdown of the keywords searched and the volume of results: "Eating Disorders" keyword search results were 43,611. "Substance Use Disorder" keyword search results were 290,129. "Anorexia Nervosa" keyword search results were 16,720. "Bulimia Nervosa" keyword search results were 7,716. "Binge Eating Disorder" keyword search results were 4,463. And "Alcohol Use Disorder" keyword search results were 106,541.

Only research articles related to human studies that were published since 1995 in the English language were included in this study. All types of research articles were included except for books and documents. After the manual screening of each article, the relevant research studies for this literature review were selected. A total of 36 articles were selected for this study to determine the risk of substance use among adolescents and adults with eating disorders.

Discussion

The results of the study showed SUD is commonly associated with EDs. There are many reasons for this comorbidity, such as common socio-demographics, neurobiological mechanisms, personality features, and environmental and genetic factors. 

Socio-demographics 

EDs are more common in women than in men. Women are more likely to report repeated body checking habits, dieting, purging and weight loss. Men are more likely to report binge eating and excessive workouts [8]. Men are 1.3 times more likely to have SUD compared to women. Although the rates of SUD are greater in men, women have higher rates of specific substances use like cocaine and psychotherapeutic drugs than men [9]. SUD is prevalent among adolescents and young adults with low income and native Americans [10]. AUD is most common among Hispanic adolescents [11].

EDs are perceived to be more common in white women than other ethnicities. Several studies were done in the past to investigate the race factor in developing EDs. Some studies found that EDs are more common among Hispanic and Latino women than white women [12]. A cross-sectional study (n = 2,554) found that any binge eating and BED are more common than AN and BN in Hispanic/Latina women. However, this could be due to the fact that Hispanic women are being more likely to exhibit binge eating habits than restricted eating habits [13]. Other studies found Black African women to be less susceptible to BED and BN. The difference in the prevalence of EDs in various race groups could be due to clinician biases such as believing EDs do not happen in minority groups or due to a lack of access to medical treatment in certain race groups [12]. BED was found to be associated with lower education. BN was the lowest among recent immigrants to the United States while the prevalence increased as the time spent increased. Generally, individuals older than thirty years are less likely to develop eating disorders [13].

Women with eating disorders are more likely to abuse substances than those with no eating disorders. Approximately 12% to 18% of adults with anorexia nervosa (AN) and 30% to 70% of adults diagnosed with bulimia nervosa (BN) have SUD [14]. One-fourth of individuals with BED reported SUD [15]. Men with BED have higher rates of SUD compared to women [16]. Several studies in adolescents found that approximately two-thirds of those with BN have used alcohol, one third had used cigarettes at least once and another third have used illegal drugs at least once. Among adolescents who used illegal drugs, marijuana was the most used drug followed by cocaine then amphetamines. Binge-purge behavior in AN and BN is associated with a high risk of tobacco, AUD and other substance use [14].

Behavioral Associations 

There are significant similarities in the behaviors of those with EDs and SUD. The binging and purging behavior in BN and BED is associated with a high risk of development SUD. Behaviors of women with AN and restriction types of eating disorders are less associated with SUD. Abusing certain substances such as alcohol is highly associated with EDs. In one study 30.1% of women who presented for treatment from AUD were diagnosed with EDs [17]. Alcohol consumption functions as a primary trigger for binge eating in women with BN. Alcohol can be used by women with EDs to suppress their appetite or to avoid and restrain from food as a compensatory behavior [17]. Attempts to restrict alcohol intake (because alcohol is high in calories) in patients with BN are usually followed by binge drinking [18]. A self-report study (n = 176) in women with BN found that dietary restriction is associated with binge drinking habits rather than frequency of drinking [19]. Individuals with EDs use alcohol as a way to cope with their eating problems. The presence of addictive personality traits could be the reason for the frequent association of AUD with EDs. EDs are frequently conceptualized as addictive disorders [20].

Women with BN are more likely to report negative consequences from the same frequency and amount of using alcohol than others without BN [21]. AUD in adolescents with BN is associated with an increase in risk-taking behaviors such as attempting suicide and risky sexual behavior [20]. SUD and BN are associated with emotional dysregulation, novelty-seeking traits, and impulsivity [22, 23]. A comparative study (n = 41) in women found that binge eating and heavy alcohol use happen separately in situations that included pleasant events, social pressure and interpersonal interactions. Both binge eating and heavy alcohol use co-occur in events associated with unpleasant emotions, physical discomfort, urging and tempting situations [24]. 

Table 2 summarizes some of the important studies that explain the behavioral association between SUD and EDs.

**Table 2:** Summary of some of the studies used to explain the behavioral association between SUD and EDs EDs: eating disorders, SUD: substance use disorder, BN: bulimia nervosa, AN: anorexia nervosa, PTSD: post-traumatic stress disorder

Author name	Year of publication	Study design	Sample size (if applicable)	Conclusions
Cohen et al. [17]	2011	Randomized Controlled Trial	122	Women who reported binge eating in the PTSD/SUD group had higher psychopathology and responded less favorably to treatment than those without binge eating.
Gregorowski et al. [18]	2013	Review	N/A	SUD is prevalent among individuals with EDs especially those with bulimic features. EDs and SUD are associated with common genetic and environmental risk factors. Laxatives, stimulants, thyroid replacement hormones can be misused as weight loss aids.
Steward et al. [19]	2000	Self-report	176	Diet restriction in BN is associated with binge drinking patterns rather than frequent drinking.
Birch et al. [24]	2006	Comparative study	41	Binge eating and heavy alcohol use happen separately in situations involved with pleasant events, social pressure and interpersonal interactions. And they co-occur in events associated with unpleasant emotions, physical discomfort, urging and tempting situations.
Anzengruber et al. [25]	2006	Observational study	1,524	Smoking is prevalent in women with BN and in AN - purging and binging type.

Caffeine and tobacco use are common in women with EDs because they can be used as an appetite suppressant. Tobacco can be used as an appetite suppressant and as a distractor from thinking about food [18]. An observational study (n = 1524) found that smoking is prevalent in women with BN and in AN - purging and binging type [25].

Studies found amphetamine use is higher among women with AN than those with BN and it is more associated with dieting and purging than binging behaviors. Similarly, cannabis and opiates use are also common among women with EDs especially among those with AN [18]. Laxatives are one of the most common substances used to lose weight with a 75% prevalence among women with EDs [18]. Studies found women with laxatives abuse are at high risk to have comorbid psychopathology. Also, laxative abuse predicts high perfectionism traits and avoidant personality disorder in this group. Diuretics are used as a method for purging in BN which causes severe electrolyte imbalance [26]. The use of different forms of purging predicts the severity of the eating disorder while the frequency of purging predicts the presence of psychopathologies such as personality disorders, depression, and anxiety disorders [27]. Diet pills are usually associated with diuretics but are less common. Abuse of thyroid replacement hormones is common to increase metabolism and enhance weight loss which causes cardiovascular and blood pressure adverse effects. Those with diabetes mellitus and EDs might reduce insulin use, especially after binge eating to help in weight loss [18].

Etiological Factors 

Environmental factors: Cumulative childhood trauma can impact normal development and causes psychopathology which includes EDs and SUD. A study by Baker and colleagues found that there is an association between childhood sexual abuse and the development of comorbid BN and SUD [28]. A study by Corstorphine and colleagues (n = 102) found an association between a history of childhood sexual abuse, SUD, and impulsivity in patients with EDs [29]. Other factors that impact the risk of development of SUD and EDs include poor paternal education, close maternal relationship, SUD or EDs behavior modeling, and maternal concern about weight loss and appearance [18].

Genetic factors: Several studies examined the possible genetic link in EDs and SUD. A study by Kendler and colleagues (n = 1030) in female twin pairs found that the development of BN and AUD are influenced by separate genetic factors. Comorbid BN and AUD might share common genetic, environmental, and familial factors [30]. A study by Slane and colleagues (n = 292) found a significant overlap in genetic factors between binge eating and compensatory behaviors in BN, and AUD. The study suggested there is a hereditable link in these two disorders [31]. A study by Redgrave and colleagues (n = 217) found that patients with EDs who had first degree relatives with AUD are more likely to use alcohol than those without. The study concluded that having first degree relative with AUD was likely to exacerbate the ED than to cause it [32].

Addiction Model and Neurobiological Factors

The addictive process involves dysfunction in three domains: the motivation and reward system, affect regulation, and behavioral inhibition [18]. SUD and BED share some addictive diagnostic criteria such as binging more amounts than intended, continuous binging despite the presence of negative consequences, and not engaging in pleasurable activities due to binging. Patients with both disorders struggle with shame and guilt after binging [15].

Both SUD and EDs share a common neurobiological process that includes disturbances in neurotransmitter functions such as dopamine, serotonin, endogenous opiates, and gamma amino-butyric acid [18]. Dopamine is involved in the motivation and rewarding process. Substance use and food rich in sugar and fat (highly palatable food) increase the dopamine levels and enhance stimulus-reward association. This increases the addiction potential. Tryptophan is an essential component of serotonin. Low levels of tryptophan are associated with high carbohydrates intake. Low serotonin levels cause a decrease in inhibitory control over food and an increase in drug cravings. Glutamate is involved in food regulation intake and substance abuse behavior. High food intake results in blockage of the glutamate receptors in the nucleus accumbens [15].

Psychopathology 

Women with comorbid EDs and SUD often present with other disorders. They present with anxiety and major depressive disorder (MDD) and post-traumatic stress disorder (PTSD). A comparative study (n = 3006) found that PTSD and MDD were mediating factors for the co-occurrence of EDs and SUD [17, 33]. Attention deficit hyperactivity disorder (ADHD) might be another mediating factor for the development of comorbid EDs and SUD. This is due to the common underlying impulsivity and the dysfunction in the neurobiological reward system in ADHD, EDs, and SUD [18]. Individuals with EDs have difficulty shifting tasks due to poor cognitive flexibility and high rigidity [34].

A study by Thomson-Brenner et al. found that there are broad personality subtypes in adolescents which make them more liable to develop EDs such as having emotional/behavioral dysregulation, being avoidant or insecure, and constricted or obsessional [35]. The onset of SUD in patients with ED is strongly associated with behavioral dysregulation which is a very common feature in patients with BN [35]. Patients with BN and AN usually have a high level of neuroticism. Patients with AN are more likely to be introverts while those with BN are more likely to have anti-social traits and high impulsivity [36].

Study Limitations

Our study is based on reviewing research articles published after 1994 and does not include possible important contributions from studies published prior to that. A systematic review in our study was not performed and no quality assessment of the selected research studies was done.

## Conclusions

The most common EDs are BN, AN, and BED. The prevalence of EDs is low in the population. BED is the most common eating disorder. EDs are more common in women than in men. SUD especially AUD is commonly associated with EDs. AUD is more associated with binge eating behavior than food restriction. Patients with BN drink alcohol excessively to distract them from thinking of food or as a way to cope with their eating disorder challenges or to suppress their appetite. Childhood trauma plays a significant role in the risk of developing EDs and SUD. Impulsivity, emotional dysregulation, and novelty-seeking traits are common features in comorbid SUD and EDs. There is a common genetic, neurobiological, and addiction model in SUD and EDs. All of these factors significantly increase the risk of SUD in adolescents and adults with EDs.

New updated screening tools should be introduced to carefully screen for substance use, especially for AUD in patients with EDs. Treatment programs for SUD should be well equipped to treat comorbid EDs. This will improve the efficacy of the whole addiction treatment process for SUD and EDs. Further research studies are needed to explore the genetic aspect of comorbid SUD and EDs.
